# CT angiography and MRI of hand vascular lesions: technical considerations and spectrum of imaging findings

**DOI:** 10.1186/s13244-020-00958-4

**Published:** 2021-02-12

**Authors:** Alain G. Blum, Romain Gillet, Lionel Athlani, Alexandre Prestat, Stéphane Zuily, Denis Wahl, Gilles Dautel, Pedro Gondim Teixeira

**Affiliations:** 1 Service D’imagerie Guilloz, CHRU Nancy, 54 000 Nancy, France; 2 Department of Hand Surgery, Plastic and Reconstructive Surgery, Centre Chirurgical Emile Gallé, CHRU de Nancy, 54 000 Nancy, France; 3 Vascular Medicine Division and Regional Competence Center for Rare Vascular and Systemic Autoimmune Diseases and Vascular Medicine Division, CHRU Nancy, INSERM UMR-S 1116 University of Lorraine, 54 000 Nancy, France

**Keywords:** Occupational disease, Thromboangiitis obliterans, Buerger’s disease, Glomus tumour, Vascular tumour

## Abstract

Vascular lesions of the hand are common and are distinct from vascular lesions elsewhere because of the terminal vascular network in this region, the frequent hand exposure to trauma and microtrauma, and the superficial location of the lesions. Vascular lesions in the hand may be secondary to local pathology, a proximal source of emboli, or systemic diseases with vascular compromise. In most cases, ischaemic conditions are investigated with Doppler ultrasonography. However, computed tomography angiography (CTA) or dynamic contrast-enhanced magnetic resonance angiography (MRA) is often necessary for treatment planning. MR imaging is frequently performed with MRA to distinguish between vascular malformations, vascular tumours, and perivascular tumours. Some vascular tumours preferentially affect the hand, such as pyogenic granulomas or spindle cell haemangiomas associated with Maffucci syndrome. Glomus tumours are the most frequent perivascular tumours of the hand. The purpose of this article is to describe the state-of-the-art acquisition protocols and illustrate the different patterns of vascular lesions and perivascular tumours of the hand.

## Key points

- Vascular lesions of the hand are common and are distinct from vascular lesions elsewhere.
- At the level of the hamatum, the ulnar artery is located in front of the tip of the hamulus, where it is exposed to shocks on the palm of the hand.
- Computed tomography angiography of the hand is performed with two successive acquisitions after injection of iodinated contrast medium.
- An artery occlusion may be missed with magnetic resonance imaging if magnetic resonance angiography is not performed.

## Introduction

Vascular lesions of the hand are common and are distinct from vascular lesions elsewhere because of the terminal vascular network in this region, the hand’s frequent exposure to trauma and microtrauma, and the superficial location of the lesions. Vascular lesions in the hand may be secondary to local pathology (e.g. tumour, malformation, trauma, iatrogenic cause, or drug injection), a proximal source of emboli (e.g. dissections or aneurysms), or systemic diseases with vascular compromise (e.g. rheumatic and vaso-occlusive diseases).

Clinical history and direct observation remain the best approach for diagnosis of vascular lesions in the hand. Symptoms may suggest emboli, vessel occlusion, a vascular malformation, or a tumour. In most cases, Doppler ultrasonography (US) is the first imaging modality to be performed. Computed tomography angiography (CTA) or magnetic resonance imaging (MRI) is indicated to improve lesion characterisation and treatment guidance [1, 2]. MRI protocols should in most cases not only include conventional sequences but also contrast-enhanced magnetic resonance angiography (MRA), which improves the evaluation of the vascular network and helps to characterise vascular malformations [3–5]. MRI is also the imaging method of choice for the evaluation of vascular tumours and the determination of their anatomic extent [6, 7]. CTA is versatile and most often used for the evaluation of traumatic lesions and vascular occlusion [2, 8, 9]. Recently, the clinical availability of advanced CT imaging techniques, such as dynamic CTA and super-high-resolution (SHR)-CTA, has increased. Dynamic CTA provides a functional analysis of the hand arterial network and warrants lesion evaluation at the best vascular phase [10, 11]. SHR-CTA allows a clear visualisation of the most distal arteries.

In this article, we will review the relevant vascular anatomy, describe the state-of-the-art acquisition protocols, and illustrate the different patterns of vascular lesions of the hand in adults, while avoiding common diagnostic pitfalls.

## Relevant anatomy

**Fig. 1:**
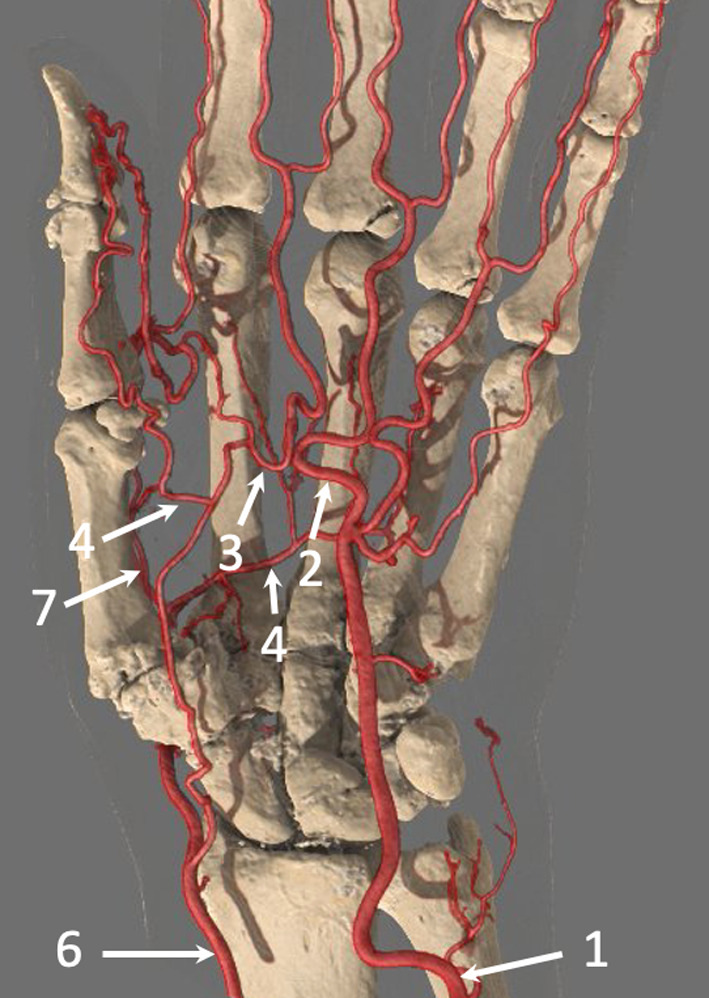
SHR-CTA of the hand (with GI) in a 69-year-old woman that shows the complex arterial network of the hand with a Type A incomplete SPA. The UA (1) feeds the ulnar side of the SPA (2), while the superficial branch of the RA feeds the radial side of the SPA (3). The SPA gives off branches into the first web space, which supply the radial side of the index finger and the thumb (4). The DPA (5) is constituted mainly from the RA (6), which forms an anastomosis with the deep branch of the UA. It generates the FPMA (7). Note the close relationship between the UA and the hamulus. Note also carpal degenerative changes and joint calcifications due to calcium pyrophosphate deposition disease

The vascularisation of the hand is mainly ensured by the radial and ulnar arteries (RA and UA, respectively) [12]. The UA penetrates the palm of the hand through Guyon’s canal in which it travels. Guyon’s canal contains the UA and its two (sometimes three) satellite veins as well as the ulnar nerve on the ulnar side. These structures are well protected from trauma at the level of the pisiform. However, at the level of the hamate, the UA is located in front of the tip of the hamulus (hook of the hamate), where it is exposed to shocks on the palm of the hand (Fig. 1; Additional file 1: Video 1) [13, 14].

The RA and the UA feed several arches that are responsible for the vascularisation of the carpus; the main ones are the superficial palmar arch (SPA) and the deep palmar arch (DPA). The SPA is mainly fed by the UA and ensures at least the vascularisation of the last three or four fingers. The SPA is covered by skin and the palmar fascia. Its most distal part projects between the proximal and distal palmar creases.

**Fig. 2:**
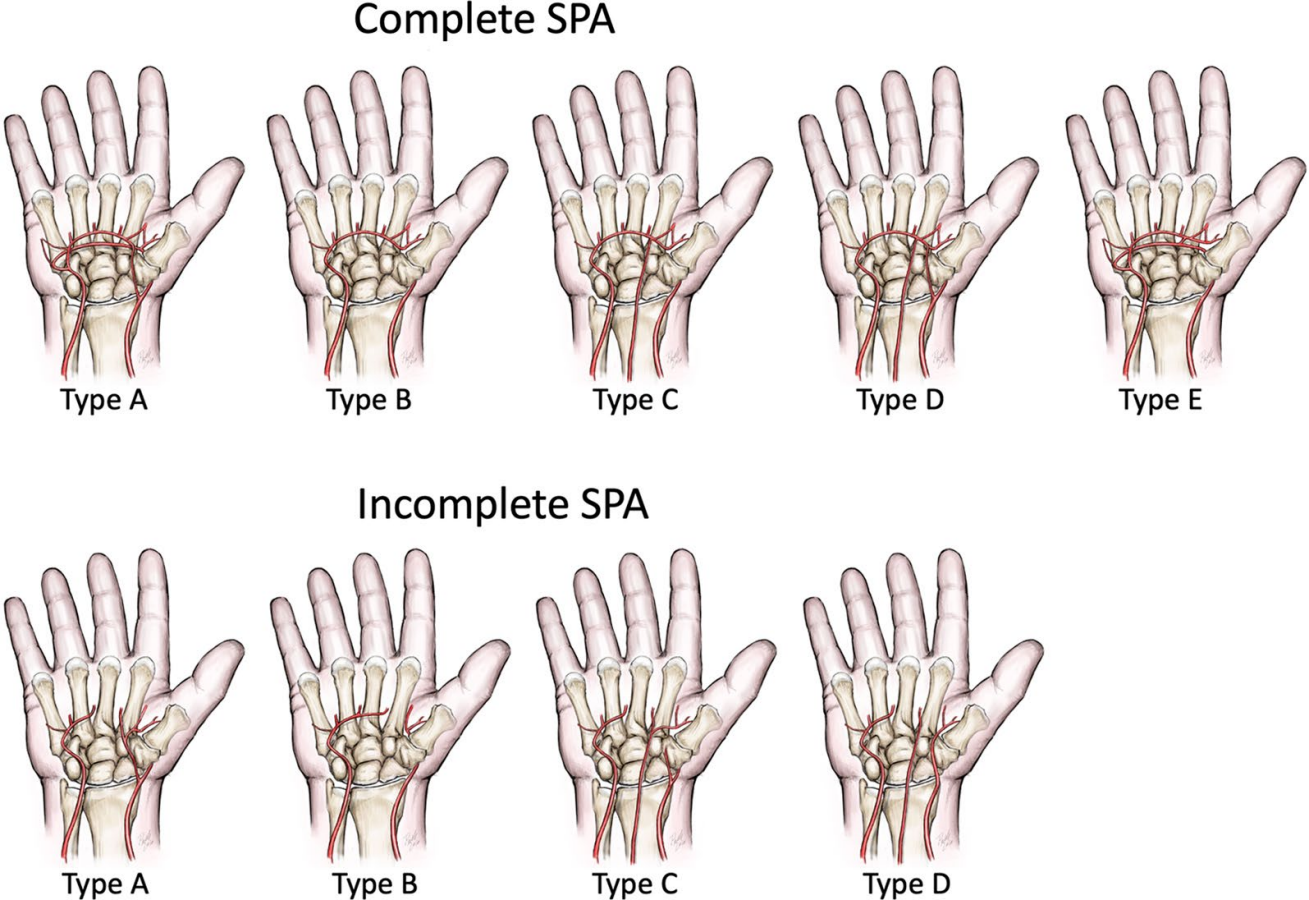
The SPA classification by Coleman and Anson [12] with two groups: group I with complete arch and group II with incomplete arch. In group I, contributing vessels anastomose with each other or the UA extends to the thumb. Group I is further divided into five types: Type A, classical radio ulnar arch, formed by the superficial palmar branch of RA and the main stem of the UA; Type B, arch formed entirely by the UA; Type C, median-ulnar arch, composed of the UA and an enlarged median artery; Type D, radio-median-ulnar arch, three vessels enter into formation of the arch; and Type E, arch initiated by the UA and completed by a large-sized vessel derived from the deep arch. In group II, contributing vessels do not anastomose with each other or the UA fails to reach the thumb. Group II is further divided into four types: A, B, C, and D, similar to group I (except Type E, which has no representation in group II). Some other subtypes have also been described. Types A and B of group I represent 70% of the cases

There are many anatomical variations of the SPA (Fig. 2) [12, 15]:

- The SPA may or may not vascularise the thumb.
- A persistent median artery is observed in about 10% of cases; its size is extremely variable. It may be barely perceptible or, conversely, equal to the diameter of the RA. Its origin is also variable. It is often associated with a bifid median nerve; in that case, it travels between the two nerve trunks [16].
- The SPA can be supplied by the RA and/or the persistent median artery.

The DPA is mainly supplied by the RA and ensures vascularisation of the first two fingers. The DPA travels to the palmar surface of the bases of the metacarpal bones deep to the deep flexor tendons. It gives rise to four palmar metacarpal branches. The first palmar metacarpal artery (FPMA) ensures vascularisation of the thumb and feeds the palmoradial digital artery of the index finger. The palmar metacarpal arteries of the second, third, and fourth spaces each receive a perforating branch originating from the corresponding dorsal interosseous artery before anastomosing with the corresponding common digital artery originating from the SPA. These arteries are then divided into palmar collateral arteries.

The thumb arterial system may be supplied by the DPA, the SPA, and the dorsal system (Fig. 1). The FPMA and the first dorsal metacarpal artery are the main branches that provide the thumb vasculature. The FPMA is dominant in 65% of cases. The palmar arteries of the thumb are almost always present and large, unlike the inconsistent dorsal arteries that have a small diameter. In most cases, the widest artery is the ulnopalmar digital artery [17]. The fingers are vascularised by two palmar and two dorsal arteries. The palmar arteries are more important. The dorsal arteries of the long fingers only vascularise the dorsal aspect of the first phalanx.

## Imaging protocols

CTA and MRI are both efficient methods to evaluate hand vessels and lesions with a vascular origin. However, advanced CT techniques, such as dynamic CTA and SHR-CTA, have increased the frequency with which CTA is performed (Table 1). In any case, to guarantee a high-quality examination, hand imaging should be carried out independently and not as part of a complete upper limb imaging protocol. For both CTA and MRA of the hand, it is important to avoid patients with cold hands and those who smoked tobacco or cannabis prior to the examination because these conditions can generate vasoconstriction, which is detrimental to the exploration of the digital arteries. Hand warming or 1 min of squeezing exercises results in physiologic vasodilation, which is useful to improve visualisation of small vessels [8, 18].

**Table 1:** Comparison of different imaging methods (except ultrasonography) to evaluate hand vessels

Imaging methods for hand vessel analysis	Angiography	Classic CTA	Dynamic CTA	SHR-CTA	MRA
Injection site	Intra-arterial	Intravenous	Intravenous	Intravenous	Intravenous
Spatial resolution	+++	++	++	+++	variable
Temporal resolution	+++	+	+++	+	++
3D analysis	+++	+++	+++	+++	++
Bone and soft tissue evaluation	+	++	+++	+++	+++

(A) CTA

**Fig. 3:**
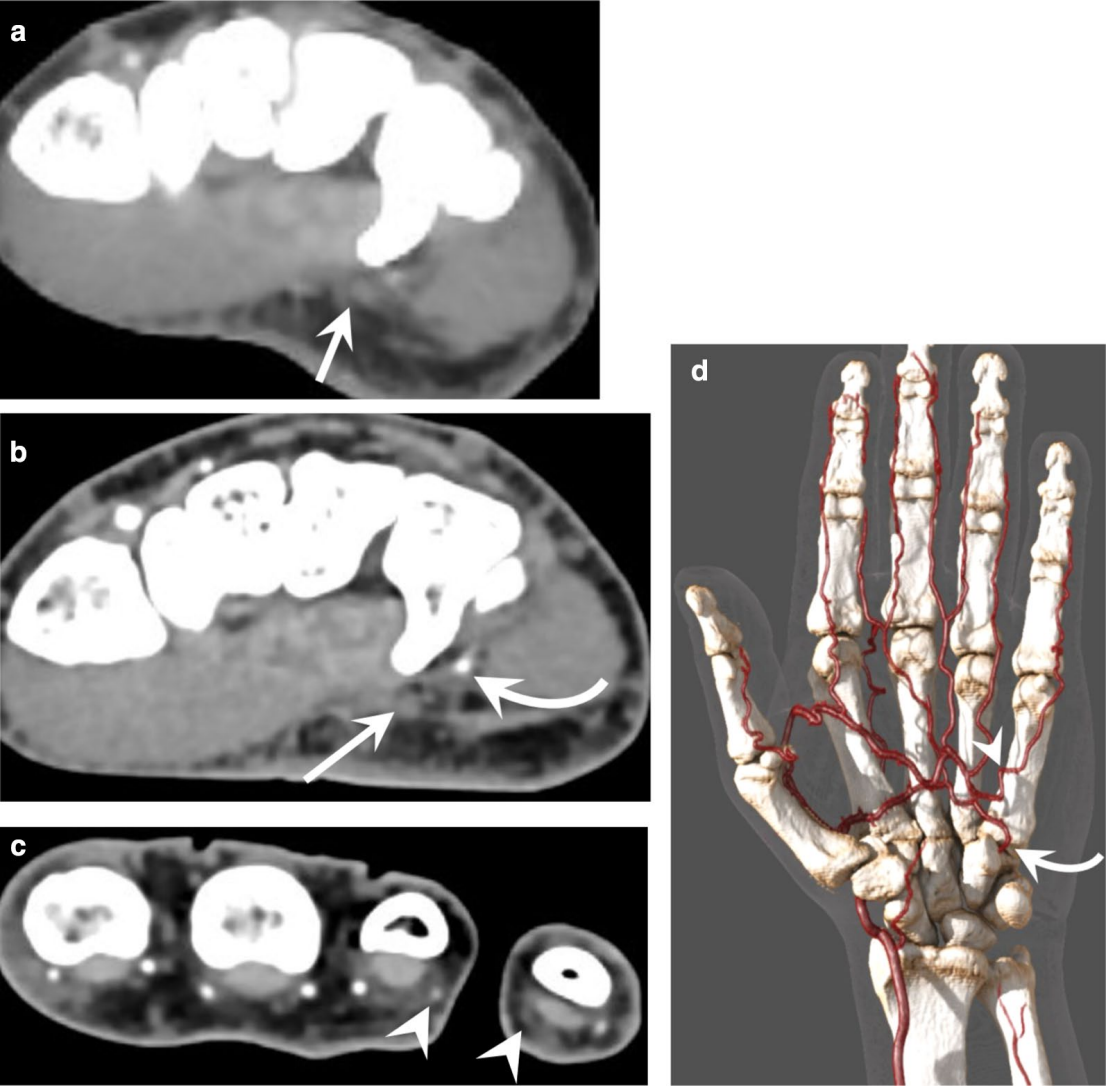
Comparison of the image quality between a complete upper extremity run-off and a CTA of the hand in a 52-year-old patient with HHS, presenting with ischaemia of the fourth and fifth fingers. **a** A complete upper extremity run-off was first obtained showing a probable occlusion of the UA. **b**, **c** CTA of the hand showed a 6-cm-long occlusion of the UA, the patency of the deep branch of the UA (curved arrow), which is fed by the DPA, the occlusion of the ulnopalmar artery of the fifth finger and the distal occlusion of the radiopalmar artery of the fourth finger (arrowheads). **d** GI providing an overview of the lesions and of the arterial configuration of the hand showing the deep branch of the UA (curved arrow) and the occlusion of the ulnopalmar artery of the fifth finger (arrowhead)

Single scan upper limb CTA can be performed with sufficient quality up to the distal portion of the RA and UA but no further. This is because it is difficult to estimate the ideal timing to achieve satisfactory enhancement of both the proximal and distal arteries and digital artery evaluation requires optimised spatial resolution, which is not achievable with an upper limb run-off (Fig. 3).

**Fig. 4:**
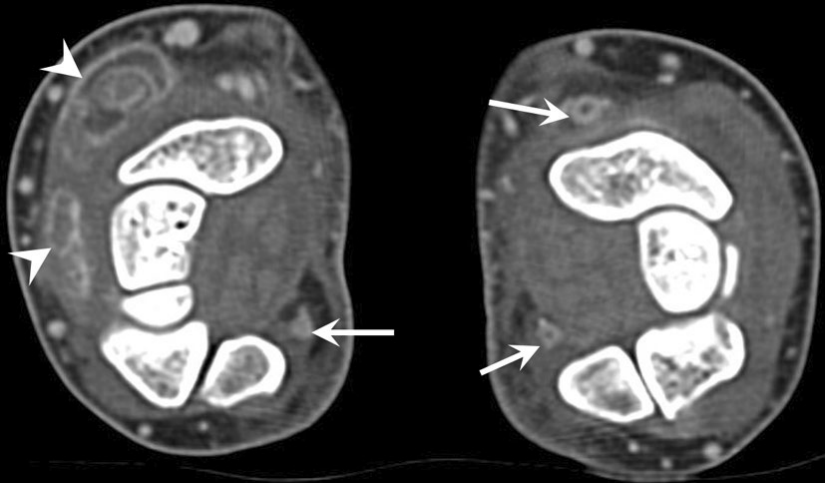
Buerger’s disease in a heavy smoker 41-year-old woman presenting with ischaemic ulcers of the fingers and arthralgia. Bilateral CTA shows occlusion and wall thickening of the right RA and of both UA (straight arrows). Note also a tenosynovitis of the extensor carpi radialis and of the extensor digitorum of the right wrist (arrowheads)

CTA of the hand if possible is performed in prone position with flat hand on the table with fingers extended (superman position) in order to place the hand as much as possible in the centre of the scanner to increase spatial resolution (Figs. 1 and 3). A bilateral exploration can occasionally be performed with the patient kneeling on the table, elbows slightly flexed, forearms resting on table, and hands together in the prayer’s position (Fig. 4; Table 2).

**Table 2:** Protocols for CTA of the hand

Scan protocols	Classic helical CTA	Dynamic CTA	Helical CTA with SHR
Precautions before scanning	Avoid cold and smoking; warm the hand; exercise by squeezing a ball		
Hand position	Prone position, hand as close as possible to the centre of the gantry		
Acquisition direction	Cranial–caudal	Not applicable	Cranial–caudal
Number of acquisitions after ICM injection	2	8–12	2
Delay between each acquisition	12 s	5 s	15 s
Scanning range	20–25 cm	4–16 cm	20–25 cm
Detector coverage and slice thickness	80 × 0.5 mm	80–320 × 0.5 mm	160 × 0.25 mm
Pitch	Detail (0.637)	0	Detail (0.569)
Scanning time for each acquisition	10 s	0.5 s	13.5 s
Scanning FOV	Ideally < 12 cm	Ideally < 12 cm	Ideally < 12 cm
Focal spot	Small	Small	Ultra-small
Matrix size	512 × 512	512 × 512	1024 × 1024
kVp	100	80	100
mAs	70–130	75	100–200
Rotation time	0.5 s	0.5 s	1 s
CM amount	1.2–1.3 ml/kg body weight		
Saline flush	30 ml		
Flow rate	4–5 ml/sec		
Arterial scanning delay	Manual bolus tracking	Test bolus or manual bolus tracking	Manual bolus tracking
Reconstruction algorithms	Iterative or DLR reconstruction with standard and high-resolution kernels		
Post-processing	MPR, MIP, GI, bone subtraction		

Optimal injection and acquisition parameters are critical when imaging the hand [19]. Iodinated contrast medium (ICM) should be injected into a contralateral peripheral vein. Alternatively, injection can also be performed in an ipsilateral antecubital vein, more proximal relative to the study area. Around 1.2–1.3 ml/kg body weight of ICM, with a concentration of at least 350 mg I/ml, is injected with a flow rate of 4–5 ml/s followed by a 30-ml saline flush. A test-bolus or bolus-tracking technique is mandatory. For helical CTA, a simple and efficient approach is to use bolus tracking with UA and RA visual monitoring and manual triggering.

In helical mode, two successive acquisitions are carried out after ICM injection, in the same direction from the proximal to distal location. Even with a delay after contrast injection over 10 s, the contrast bolus can be outrun during the first acquisition. A double acquisition allows a better distinction between arteries and veins, as well as between delayed opacification and occlusion of the digital arteries. A late acquisition may also be useful for detecting arterial wall thickening and enhancement in cases of occlusion or an inflammatory arterial wall disease. A low-dose technique with 80 or 100 kVp increases the contrast at the enhanced vessels and reduces patient exposure [18].

**Fig. 5:**
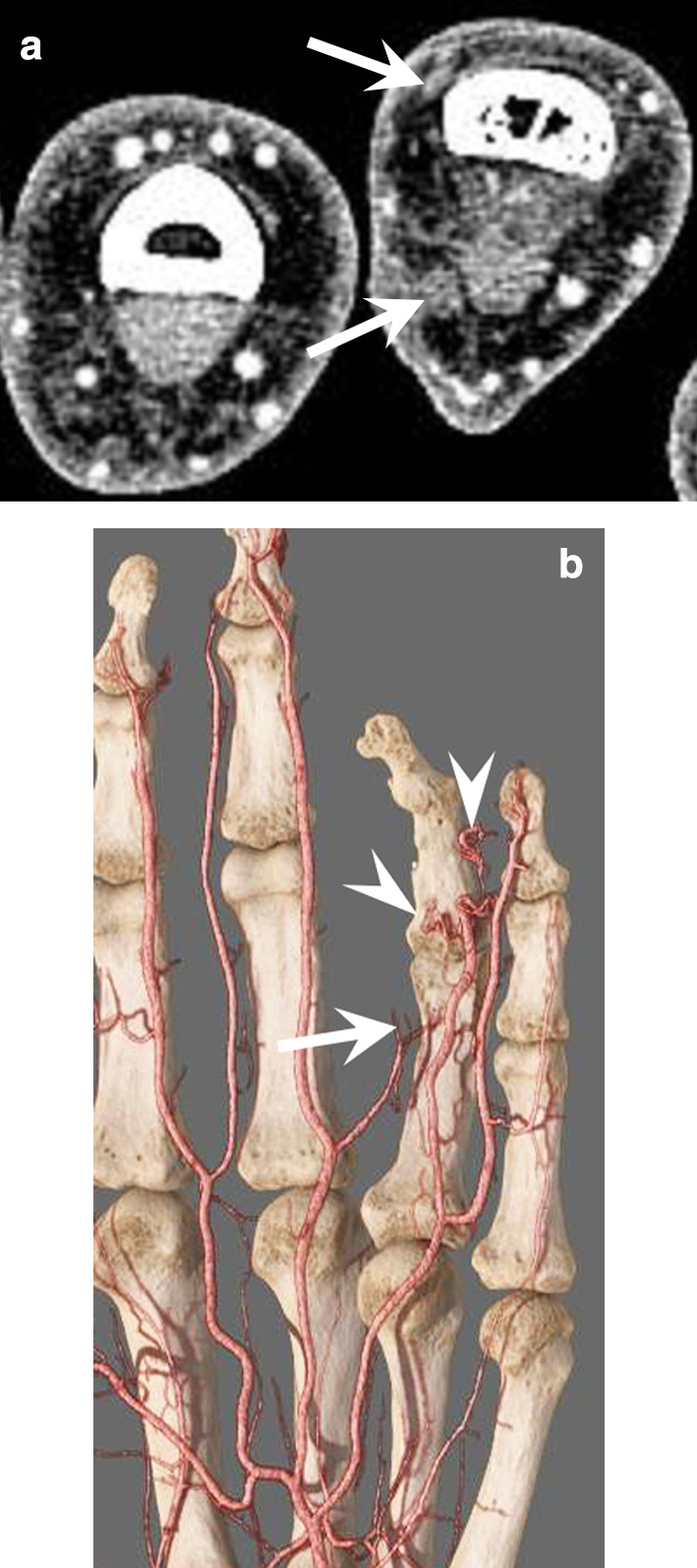
Traumatic occlusion of the radial digital arteries of the fourth finger in a 29-year-old man. **a** SHR-CTA at the level of the first phalanx showing the occlusion of the radiopalmar and radiodorsal digital arteries of the fourth finger (arrows) as well as a reduction of the venous flow. **b** GI showing the occlusion of the radiopalmar artery at the level of the first phalanx and of the ulnopalmar artery associated with corkscrew collaterals (arrowheads)

SHR-CTs are novel commercial systems with 0.25-mm detector elements in the in-plane and longitudinal directions. Images can be reconstructed with high matrix sizes (up to 2048 × 2048) using deep-learning reconstruction algorithms. SHR improves the quality of both native images and multiplane reformats, especially the depiction of small vessels, particularly the distal portion of the digital arteries (Fig. 5; Additional file 1: Video 1) [20].

**Fig. 6:**
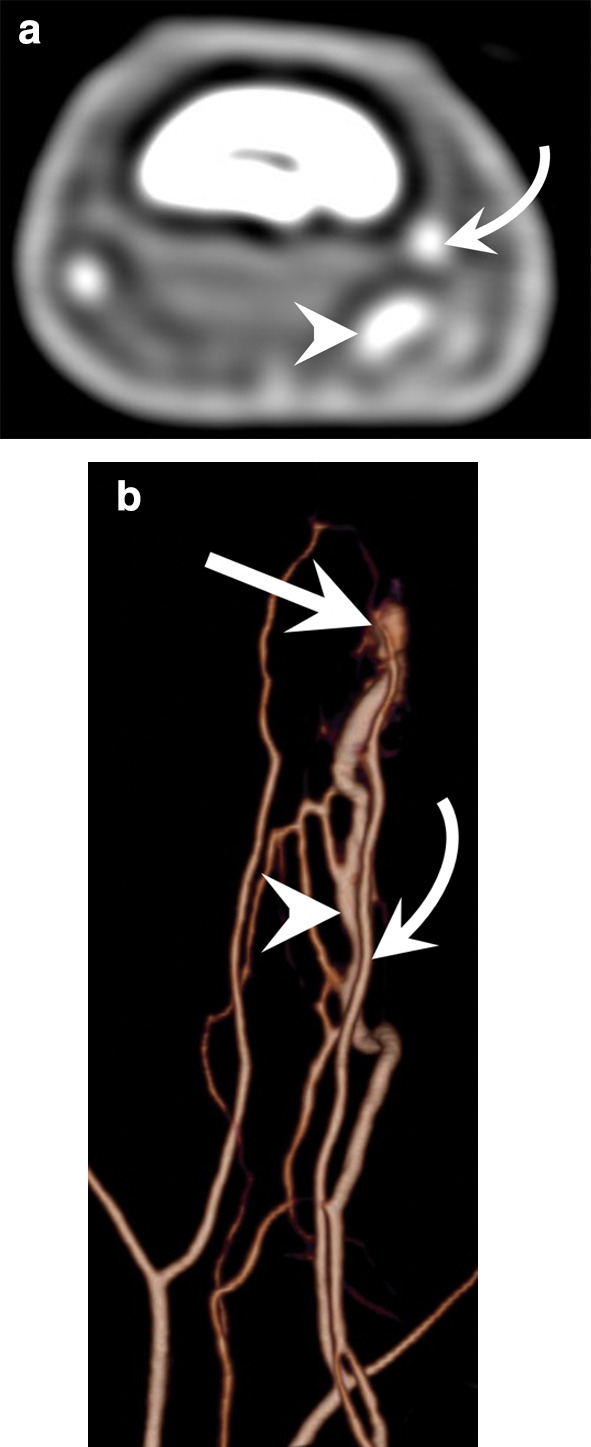
Dynamic CTA of an arteriovenous fistula (straight arrow) fed by the ulnopalmar digital artery (curved arrow) of the fourth finger in a 32-year-old patient. **a, b** Native slice and VRT image with bone subtraction at the early arterial phase showing the fistula and the early enhancement of an ulnopalmar vein (arrowhead). Dynamic CTA allows capturing the lesion at the best vascular phase

Dynamic CTA simultaneously yields an anatomic and a functional vascular evaluation [10, 11, 18]. Large detector CT scanners (e.g. CT perfusion) allow multiphasic arterial exploration with up to 16 cm of Z-axis coverage without compromising spatial or temporal resolution. Indeed, dynamic CTA warrants a short volume acquisition time (0.27–1 s) with temporal uniformity (e.g. all voxels of a given volume are acquired almost at the same time), and a free choice of inter-volume delay. A 5-s inter-volume delay is a good trade-off between temporal resolution and radiation dose. If bone subtraction techniques are applied, images with a pseudo-angiographic effect can be generated (Fig. 6). Dynamic CTA of the hand can be useful to evaluate vascular malformations because it allows visualisation of the lesion at the best vascular phase (Fig. 6; Additional file 2: Video 2). It also provides a functional evaluation of the arterial network of the hand in the preoperative evaluation of hypothenar hammer syndrome (HHS). The same technique applied at the shoulder girdle has also been shown to be effective in diagnosing thoracic outlet syndrome [21].

Post-processing is required to visualise and interpret SHR-CTA datasets and to communicate findings to referring physicians [8, 9, 22, 23]. Post-processing includes multiplanar reconstruction (MPR), maximum intensity projection (MIP) images, and volume rendering technique (VRT), which has been replaced by global illumination rendering (GI) [24, 25]. In all cases, three-dimensional (3D) images must be analysed in comparison with native slices due to possible artefacts in small vessel segmentation. This endeavour requires high spatial resolution, good vascular enhancement, and satisfactory signal-to-noise ratio to be effective.

Finally, the radiation dose to the patient remains low, ranging from 0.05 to 0.3 mSv, due to low-dose techniques and a low hand radiosensitivity at the level of the hand [11].

(B) MRI

**Fig. 7:**
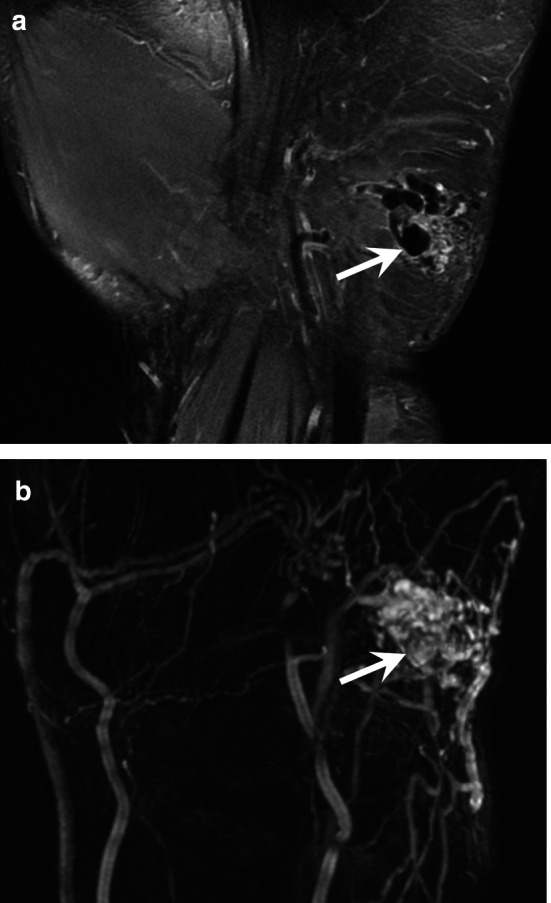
AVM of the hypothenar eminence in a 37 year-old-woman. **a** Coronal T2-weighted MR image showing the nidus with multiple enlarged vessels with flow void (arrow) indicating a high-flow malformation. **b** MRA demonstrating the high flow of the malformation with a rapid vascular enhancement of the nidus (arrow), multiple feeding arteries, and early venous drainage through dilated veins

The patient should be scanned in a comfortable position to avoid motion artefacts. A dedicated coil is essential to obtain high-quality images regardless of the field strength of the MRI system (Fig. 7). A flexible coil is used for the hand, and a wrist coil is used for the wrist or the thumb.

In most cases, MRA is not the only acquisition to be performed and the protocol also comprises conventional sequences with T1-weighted, fluid-sensitive sequences such as fat-suppressed T2-weighted MRI scans, and contrast-enhanced T1-weighted fat-suppressed sequences (Table 3). These morphologic sequences allow the characterisation of soft tissue masses and also the evaluation of the para-arterial environment (e.g. extrinsic compression, inflammatory changes). In addition, contrast-enhanced T1-weighted fat-suppressed sequences provide a means to evaluate thickening and enhancement on the arterial wall.

**Table 3:** Suggested MR imaging protocol for wrist imaging at 3 T

Planning	Type of sequence	FOV (cm)	TR (ms)	TE (ms)	Slice thickness (mm)	Matrix
Ax T1	SE or FSE	10–15	600	15	2.5–3	384 × 192
Ax T2 FS	FSE	10–15	6000	70	2.5–3	384 × 224
Cor T2 FS	FSE	10–15	6000	70	2.5–3	384 × 224
Cor MRA	time-resolved 3D sequence (TRICKS)	15	4.4–7.9	minimal	1.8–2	256 × 224
Ax T1 C + FS	SE or FSE	10–15	600	15	2.5–3	384 × 224

*Ax* axial, *Cor* coronal, *FOV* field of view, *SE* spin-echo, *FSE* fast spin echo, *TR* repetition time, *TE* echo time, *T1 C + FS* contrast-enhanced T1-weighted fat-suppressed sequence

The technical aspects and specific protocols for MRA have been well described in the literature [3, 4, 26, 27]. MRA is usually based on the administration of a gadolinium-based contrast agent and time-resolved approaches. Time-resolved sequences use various patterns of partial K-space filling with an oversampling of the K-space centre; the approach generates images with good temporal and spatial resolution compromise. Images can be analysed with subtraction technique, which improves contrast resolution, or without subtraction technique, thus allowing visualisation of the background. Although not recommended for quantitative perfusion analysis, time-resolved sequences are ideal for visual analysis of the hand vasculature and provide a vascular map and haemodynamic information, including inflow in the vessels or arrival times to the tissue [1, 3, 18]. For the evaluation of the hand, we use a time-resolved 3D sequence with a repetition time (TR) of 4.4–7.9 ms and a minimal echo time (TE min), 20–30° flip angle, a field of view (FOV) of approximately 15 cm, an approximately 224 × 256 matrix, and a 1.8–2.0-mm slice thickness. In general, an injection of 0.1 mmol/kg of contrast agent at a rate of 1.5–2 ml/second provides arterial imaging with high vessel to background contrast. The use of an injection pump is necessary, and the delay between acquisition start and contrast injection is dependent on the length of the mask acquisition, which usually lasts around 40 s (variations depending on coverage and slice thickness). For optimal evaluation of the hand, contrast injection starts 30 s after the beginning of the mask sequence.

## Different disease entities

The diagnosis of vascular lesions of the hand is often based on clinical history and clinical examination. Most of the pathologies require US examination which in many cases is sufficient to reach the diagnosis. However, CTA and MRA which provide distinct advantages over ultrasound may be indicated, and we will focus on their findings in the different entities. It should also be noted that incidental discovery of a vascular lesion on MRI is not uncommon and radiologists must be aware of this situation.

This chapter is divided into two parts. The first part covers vessel injuries and ischaemia causing lesions. The second one describes the vascular malformations and the vascular and perivascular tumours affecting the hand.

(A) Ischaemic phenomena

The causes of hand ischaemia are varied (Table 4). The three main causes of digital ischaemia, which alone represent almost two-thirds of patients with hand ischaemia, are autoimmune disorders (foremost among which is systemic sclerosis [SSc]), occupational causes, and Buerger's disease. It is likely that traumatic or microtraumatic arterial injury in the hand is widely underdiagnosed because: (1) the causative trauma is often unrecognised; (2) symptoms may be delayed, minor, or non-specific; (3) the clinical presentation may mimic other causes of digital ischaemia; and (4) imaging findings may be subtle or confusing.

**Table 4:** Causes of hand ischaemia and indication for CTA or MRA

Causes of hand ischaemia	Underlying aetiology	CTA or MRA
Peripheral emboli	Cardiac disease Thoracic outlet syndrome Atherosclerosis Aneurysm and pseudoaneurysm of proximal arteries of the upper limb	NI Dynamic CTA of the thoracic outlet CTA of the entire upper limb CTA of the entire upper limb
Iatrogenic	Radial access for monitoring or catheter-based procedures	CTA may be indicated in cases of pseudoaneurysm
Acute and subacute trauma	Lacerations, fracture, crush injury, etc	CTA
Occupational	HHS HAVS	CTA or MRA of the hand CTA or MRA of the hand
Intra-arterial injections of drugs	Voluntary or accidental intra-arterial injection of drugs into an artery in the hand or upper limb	CTA may be indicated in cases of pseudoaneurysm
Thromboangiitis obliterans	Occlusion of small- and medium-sized arteries	CTA or MRA of the entire upper limb and of the hand
Autoimmune and rheumatic diseases	Systemic sclerosis Systemic lupus erythematosus Vasculitis (PAN), Sjogren’s syndrome	Usually NI but CTA or MRA may be performed to rule out vasculitis mimics
Drug/chemical-related	Amphetamines, beta-blockers, bleomycin, cisplatin, cyclosporine, interferon, methysergide, polyvinyl chloride	NI
Vaso-occlusive disease	Cold agglutinin disease, cryofibrinogenemia, malignancy (including paraneoplastic phenomenon), etc.	NI
Other causes	Frostbite	NI

*NI* not indicated

### Peripheral embolism

**Fig. 8:**
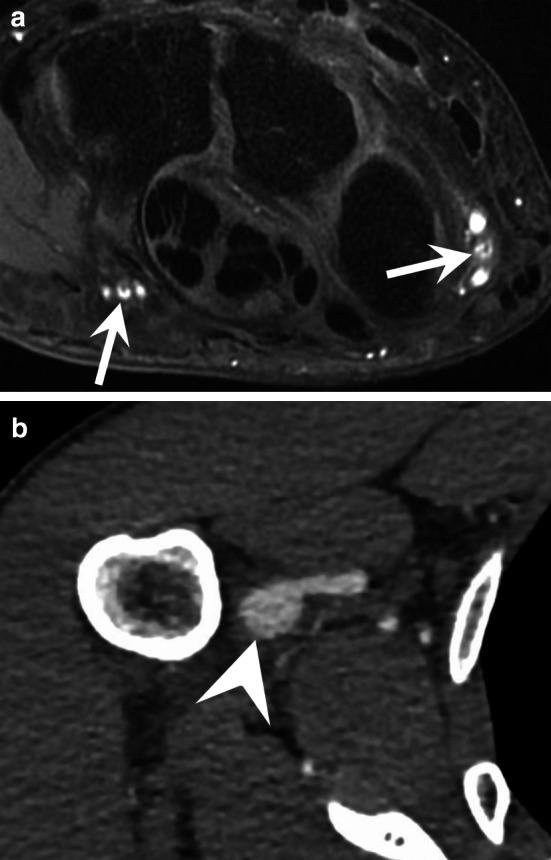
Distal emboli due to an aneurysm of the axillary artery in a 29-year-old female professional volleyball player. **a** Axial contrast-enhanced T1-weighted fat-suppressed sequence showing some emboli in the UA and RA (arrows). **b** CTA of the entire upper limb showing an aneurysm of the axillary artery

Peripheral emboli affect the upper extremities in up to 20% of cases, 70% of which are cardioembolic while 30% are caused by vascular lesions (proximal aneurysm, traumatic lesion, etc.) [28]. Therefore, imaging of the arteries of the entire upper limb is necessary in the event of suspected embolism due to lesions of the large vessels. Radiologists should specifically search for aneurysms of the axillary artery or posterior circumflex artery in sports with overhead arm motion such as volleyball and tennis (Fig. 8) [29].

### Iatrogenic causes

Radial and ulnar arterial access is frequently used for catheterisation, and transradial access is increasingly used for coronary angiography and percutaneous coronary intervention [30]. Iatrogenic conditions are mainly represented by occlusion of the RA. This phenomenon occurs in 6%–8% of cases after radial catheterisation [31, 32]. It must be prevented by appropriate anticoagulation and should be detected by US before the patient is discharged. It is generally silent, but it affects the vascular reserve of patients who may have to undergo further vascular exploration [31, 32]. Post-catheterisation aneurysm is exceptional and generally occurs in an infectious context. Iatrogenic vascular-surgery-related hand complications also include RA harvest for coronary artery bypass, haemodialysis access, or axillo-femoral bypass graft [33, 34]. Finally, pseudoaneurysm of the SPA may exceptionally occur after carpal tunnel decompression.

### Acute and subacute trauma

**Fig. 9:**
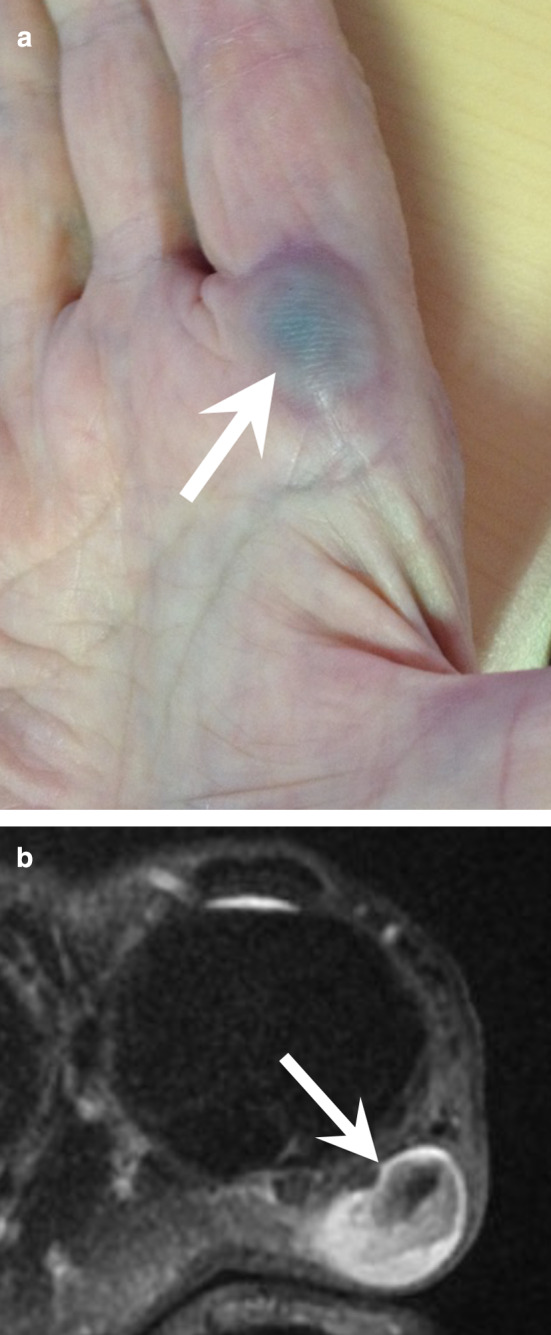
Occluded pseudoaneurysm of the radiopalmar artery of the index finger in a 79-year-old woman with repetitive microtrauma. **a** Photograph showing a blue mass under the skin at the level of the metacarpophalangeal joint (arrow). **b** Axial T2-weighted MR image showing the pseudoaneurysm (arrow) with an intraluminal thrombus

Upper extremity arterial injuries account for up to 50% of all peripheral vascular injuries. Of these, brachial artery lacerations are the most common, followed by those of the RA and UA, while digital artery lacerations are the least frequent. Digital artery lacerations usually occur secondary to open puncture wounds to the hand, but these injuries may also result from severely displaced fractures, crush injuries, gunshots, and other penetrating wounds. Acute artery lacerations of the hand do not require a CTA, except in cases of associated complex bone lesions or multiple vascular lesions. CTA findings include active extravasation, luminal narrowing, lack of luminal contrast opacification, filling defect, arteriovenous fistula, and pseudoaneurysm (Figs. 5 and 9) [22, 35–37]. Surprisingly, pseudoaneurysms of the hand are exceptional despite the high frequency of hand trauma (Fig. 9). They can affect the RA or UA, the palmar arches, and the digital arteries, presenting clinically as a small mass, sometimes occurring several months after the trauma without any pulsatility. When surgery is required, CTA or MRA provides a global assessment of the vascular network required for treatment planning [37, 38].

Occlusion of the digital arteries that results from acute blunt trauma is exceptional [18]. Those described in baseball catchers affect the index finger and are associated with hypertrophy of this finger [39]. Finger artery occlusion has also been described after prolonged strangulation of the fingers due to the wearing of a plastic bag.

### HHS

HHS is in most cases due to repetitive trauma to the UA at the level of the hamulus of the hamatum, leading to a stenosis, an occlusion, and/or an aneurysm of the UA. It is referred to as hammer syndrome because in most cases it originates from repetitive strikes and microtrauma to the ulnar side of the palm of the hand [14, 40]. However, HHS may also be due to a unique trauma on the palm of the hand.

**Fig. 10:**
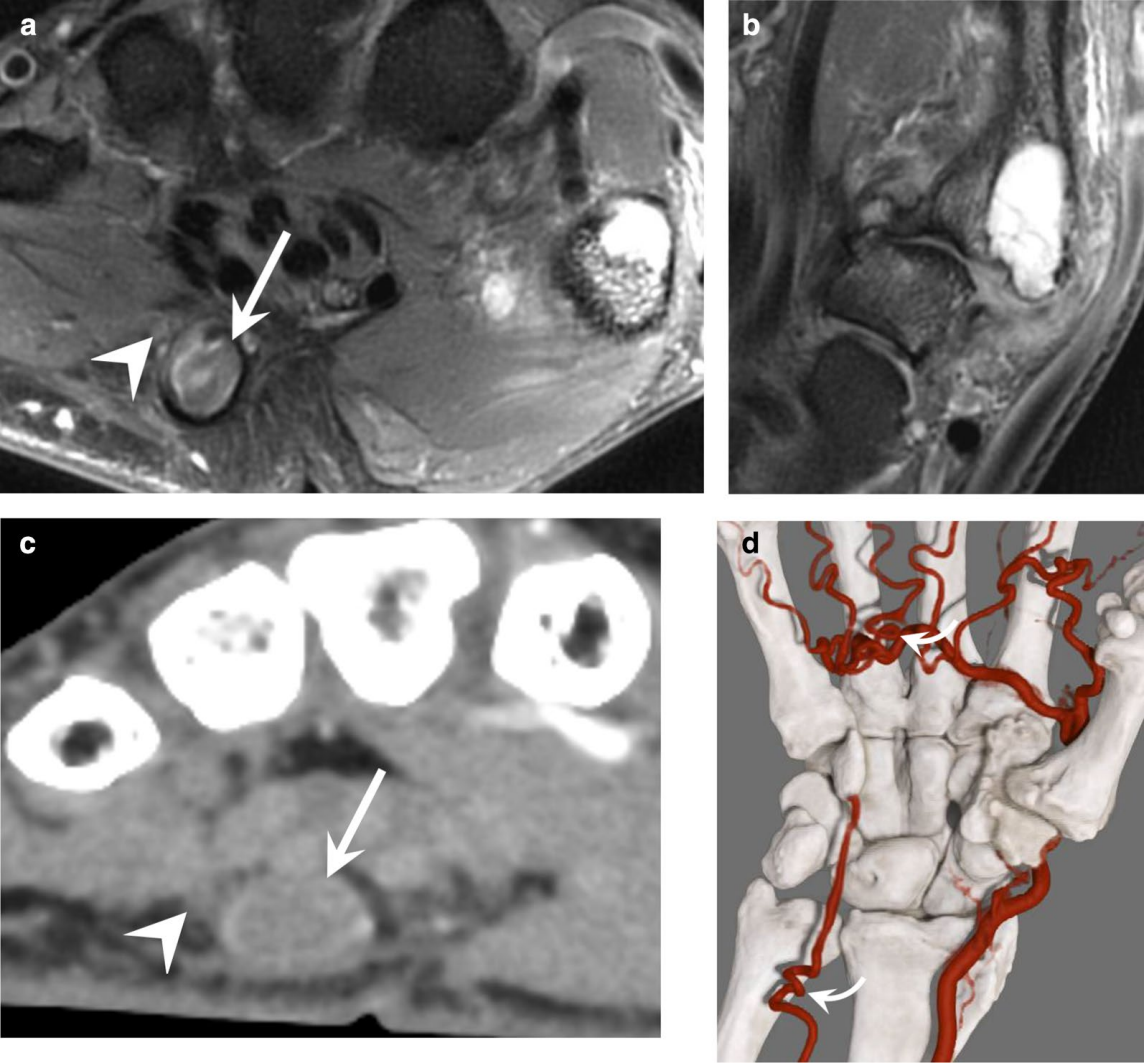
HHS in a 55-year-old manual worker exposed to different causative factors, including vibrating tools. **a**, **b** Axial and coronal T2-weighted MR images showing an occluded aneurysm (arrow) of the distal portion of the distal UA and severe osteoarthritis affecting the trapeziometacarpal joint. **c**, **d** Axial image and GI with dynamic CTA showing the distal occlusion of the UA, the patency of the SPA fed by the FPMA, a corkscrew deformity of common digital arteries, and the UA at the level of the ulnar head (curved arrow). Note the close relationship between the aneurysm and the superficial branch of the ulnar nerve (arrowhead)

The clinical manifestations depend on the location and extension of the lesions as well as the configuration of the vascular network. HHS can be asymptomatic, or it can generate Raynaud’s phenomenon and acute ischaemic manifestations with embolic digit pulp necrosis. Some clinical symptoms may be misleading because a UA aneurysm can be responsible for a mass effect or cause compression of the ulnar nerve or of its superficial (sensitive) branch (Fig. 10; Additional file 3: Video 3).

HHS is common in middle-aged, active, smoking men; it generally affects the dominant hand. HHS can be related to sports (racket sports, mountain biking, and breakdancing) or professional activities in which the hand is used to hit, push, or squeeze hard objects. HHS is recognised as an occupational disease in most countries; workers considered ‘at risk’ include metalworkers, car mechanics, lathe operators, machinists, miners, stonemasons, butchers, bakers, masons and carpenters, lumberjacks, and vibrating tool users.

**Fig. 11:**
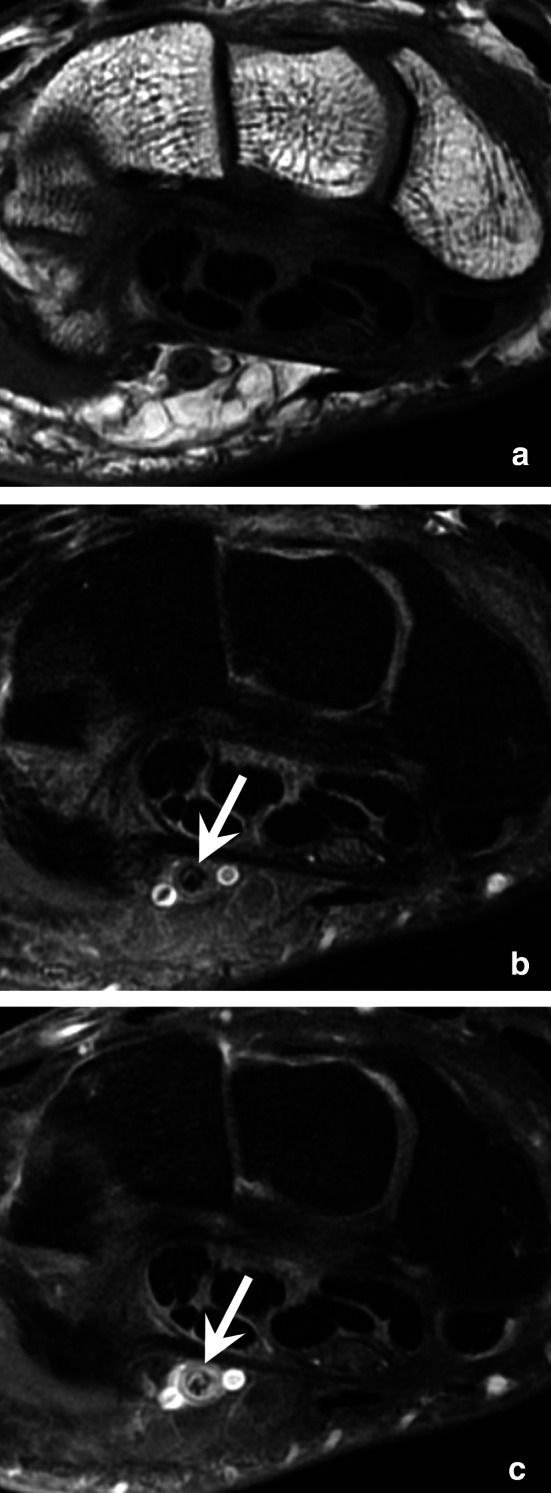
A pitfall in a case of HHS in a 45-year-old manual worker with an occlusion of the UA. **a–c** Axial T1-weighted (**a**) and T2-weighted (**b**) MR images and contrast-enhanced T1-weighted fat-suppressed sequence (**c**) showing a subtle thickening and parietal enhancement of the UA (arrow). The thrombus should not be confused with a flow phenomenon

CTA and/or MRA may be indicated to confirm the diagnosis [41, 42]. They are mainly performed if surgical treatment is discussed to show the extent of vascular occlusion and its functional impact [43]. It should be noted that the late phase with CTA and contrast-enhanced T1-weighted fat-suppressed sequence show a thickening and an enhancement of the arterial wall that underline the thrombus. For these reasons, the diagnosis may be overlooked if only conventional sequences are performed because the thrombus may be misinterpreted as a flow phenomenon (Fig. 11). UA changes initially result in parietal thickening that gradually progresses to occlusion. Occlusion then extends proximally in a variable manner towards the level of the first carpal row or even the distal ulna. Distal compromise of the SPA is also frequent. Intimal lesions may result in clot formation and emboli to the digital arteries (usually of the fourth and fifth fingers). Aneurysms are less common. Arterial dysplasia is frequently associated; it is characterised by a tortuous, helical (corkscrew) deformity of the UA, SPA, and sometimes common digital arteries [14, 18, 40]. This deformity is probably associated with arterial wall fragility and may increase the risk of arterial trauma because of greater exposure of the affected segment of the UA to the hamulus.

Surgical treatment is indicated when there is an aneurysm, ischaemic phenomena, or severe functional impairment resistant to medical treatment. It consists of removing the affected segment and reestablishing flow with venous or arterial bypass. Smoking cessation is obviously recommended, and occupational reclassification may be indicated [43–45].

A so-called thenar hammer syndrome has been described and is exceptional. Repeated microtrauma to the palmar surface of the wrist is likely to generate lesions of the RA that are identical to those described for the UA in HHS. Playing volleyball has been reported to cause this condition.

### Hand-arm vibration syndrome (HAVS)

**Fig. 12:**
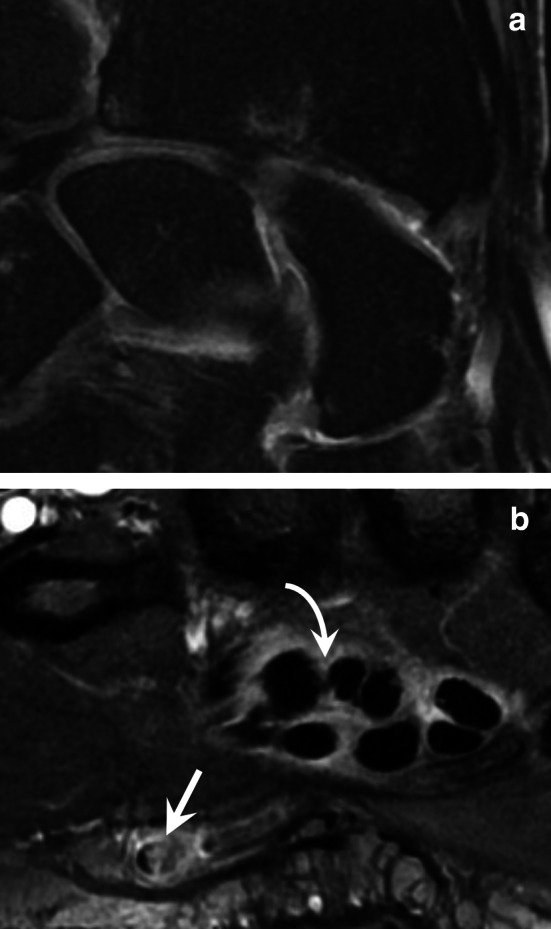
HAVS in a 62-year-old man. **a** Coronal T2-weighted MR image showing isolated osteoarthritis of the radioscaphoid compartment of the radiocarpal joint. **b** Contrast-enhanced T1-weighted fat-suppressed sequence showing a focal occlusion at the junction of the UA and the SPA (straight arrow) and a tenosynovitis of the flexor digitorum tendons (curved arrow). The UA was otherwise preserved

HAVS is a prevalent occupational disease that affects workers in multiple industries in which vibrating tools are used. The exact prevalence is unknown, but it has been estimated at 50% among exposed workers. HAVS mainly affects men over 35 years of age. It has three components: vascular, sensorineural, and musculoskeletal. Symptoms vary by intensity and duration of vibration exposure [45, 46]. Neurosensory signs appear first (paresthesia, loss of sensitivity, and carpal tunnel syndrome). Its vascular component, also known as vibration white finger, is a type of Raynaud’s phenomenon. Imaging techniques may show the vascular lesions found in HHS because HHS and HAVS share some aetiological factors. Imaging techniques may also show carpal osteoarthritis, carpal bone cysts, tendinopathies, and osteonecrosis of the lunate (Kienböck’s disease) (Fig. 12) [45, 47].

HAVS is substantially underrecognised. This underdiagnosis is problematic because early recognition and management of this condition are crucial for preventing progression and improving prognosis. Management involves reduction of vibration exposure, avoidance of cold conditions, smoking cessation, and medication.

### Persistent median artery thrombosis

**Fig. 13:**
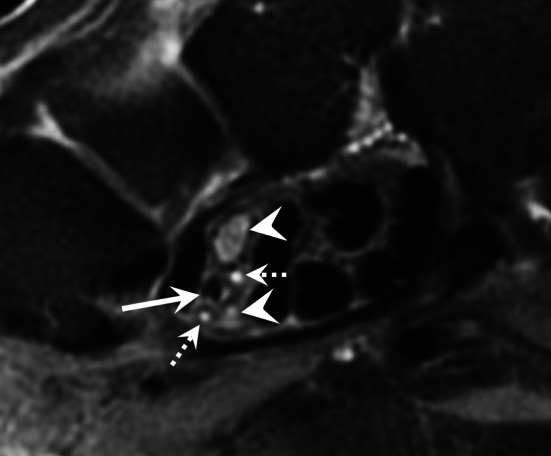
Persistent median artery occlusion in a 48-year-old woman. Axial T2-weighted MR image showing an occlusion of the persistent median artery (straight arrow) and a bifid median nerve (arrowheads). Note also the small median artery satellite veins (dotted arrows)

The persistent median artery may be the site of an occlusion or an aneurysm that causes carpal tunnel syndrome (Fig. 13) [48]. This pathology is rare and its mechanism is unclear.

Intra-arterial drug injections.

Arterial complications result from inadvertent and often repeated arterial puncture during attempted venous access and can lead to acute vascular emergencies. Vascular complications are common in people who inject drugs. They can occur locally at the injection site or at a distant location and may be arterial or venous in nature [49]. They can lead to tissue loss and require amputation in 30% of patients [50]. Various offending agents have been described, including temazepam, flunitrazepam, zolpidem, heroin, midazolam, cocaine, and buprenorphine [51]. Crushed pills lead to a significantly higher incidence of amputation compared with pure drug substances [50]. Associated infections are common and represent a poor prognostic factor.

**Fig. 14:**
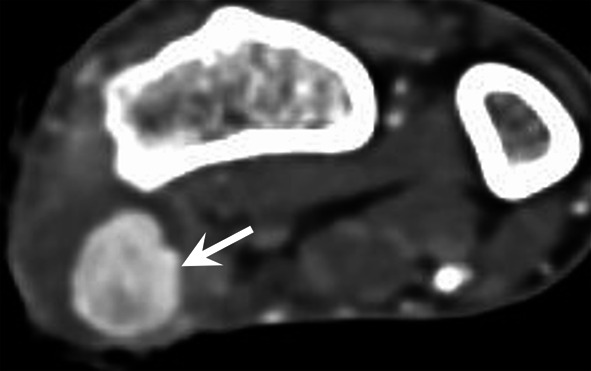
Pseudoaneurysm of the RA (arrow), after intra-arterial injection of buprenorphine, demonstrated with CTA

A multimodal imaging strategy is often required in the assessment of these vascular complications, typically involving a combination of ultrasound and CT (Fig. 14) [49]. Vascular complications may manifest as injury to the vessel wall, pseudoaneurysm or arterial thrombosis with resultant ischaemia or haematogenous spread of a pathogen from the injection site. CT is the imaging modality of choice for mycotic aneurysms. Gas within the aneurysm is a rare but characteristic sign, which is best seen on CT. Additional imaging features more commonly seen on CT include a lobulated vascular mass, an irregular and poorly defined arterial wall, peri-aneurysmal soft tissue stranding, and oedema. In addition to the risk of rupture, mycotic aneurysms may also lead to the development of arteriovenous fistulae or serve as a source of sepsis or septic emboli [49]. Thrombolysis and surgical revascularisation may be indicated to restore blood flow to ischaemic tissues in the hand.

### Thromboangiitis obliterans

Thromboangiitis obliterans, also known as Buerger’s disease, is a rare, non-atherosclerotic inflammatory disease of unknown aetiology. It typically affects young male smokers (younger than 45 years). Cannabis use has also been implicated. Although Buerger’s disease has a worldwide distribution, it is more prevalent in the Middle East and Far East than in North America and Western Europe [52].

This disease primarily affects small- and medium-sized arteries and veins in the upper and lower extremities, usually beginning in the small distal vessels, resulting in distal ischaemia. As the disease progresses, it may involve more proximal arteries. The upper limbs are affected in 25% of cases [52, 53]. Arthralgia and even arthritis are present in 12% of cases. Superficial venous thrombosis is also a typical finding.

CTA, MRA, or angiography shows involvement of the small- and medium-sized vessels in the upper (and lower) extremities, including the palmar and plantar arches, the RA and UA, and the digital arteries of the fingers (Fig. 4). The most common imaging findings are segmental occlusive lesions with bridging or corkscrew collaterals around the areas of occlusion. The disease tends to be more severe distally, with interspersed normal vessels. There should be no evidence of atherosclerosis or other proximal source of emboli (e.g. dissections or aneurysms) [53].

The cornerstone of treatment of Buerger’s disease is strict abstinence from exposure to all tobacco-containing products. Vasodilator therapy with iloprost should be considered in acute phases, and bypass surgery may be indicated for limb salvage in critical ischaemia.

### Systemic diseases and vasculitis

SSc is a rare multisystemic disease that preferentially affects middle-aged women. Hand involvement is a major feature in patients with SSc, with severe Raynaud’s phenomenon in early stages, and skin thickening. Ischaemic digital ulcers may be responsible for major disability. Although rarely indicated for the evaluation of patients affected by SSc, vascular involvement as well as bone and other soft tissue lesions can be identified on CTA or MRA.

Systemic vasculitides affecting the hand are mostly represented by polyarteritis nodosa (PAN). PAN is a systemic necrotising vasculitis that typically affects medium-sized arterial vessels but may also affect small-sized arterial vessels. PAN is usually diagnosed in middle-aged and older adults. Systemic symptoms can be the only complaints but almost every organ can be involved. Characteristic lesions of the hand include multiple short segment stenoses of the proper and common digital arteries, ectasia, and aneurysms with thickening of the vessels [3]. Similar findings have been reported for granulomatosis with polyangiitis and eosinophilic granulomatosis with polyangiitis and microscopic polyangiitis.

(B) Tumours and vascular malformations

Hand tumours are encountered frequently. Of all soft tissue lesions throughout the body, 15% are seen in the hand [54]. Lesions with a vascular origin comprise a wide and heterogeneous spectrum of injuries. The International Society for the Study of Vascular Anomalies (ISSVA) classification distinguishes between vascular tumours (lesions with cell proliferation) and vascular malformations (due to an innate disruption of vascular morphogenesis) with different pathogenesis, prognosis, and treatment [55, 56]. Vascular malformations are classified into low-flow malformations (lymphatic, capillary, or most often venous) and high-flow malformations (arteriovenous fistula and arteriovenous malformation (AVM)). The soft tissue tumours are classified according to the ISSVA and the World Health Organisation (WHO) classification of soft tissue tumours [57].

Some ambiguities still persist because some of these lesions might be classified as tumours or pseudo-tumours and malformations. In addition, tumours and malformations may also be associated. Furthermore, the current WHO classification of soft tissue tumours recognises three perivascular (pericytic) tumour types: glomus tumours, myopericytoma, and angioleiomyoma [57].

MRI provides valuable information for the assessment and treatment of these lesions. Firstly, MRI can determine the nature of many of these lesions. MRA can also characterise the flow pattern of the vascular malformations to guide treatment towards trans-arterial embolisation for high-flow malformations and percutaneous embolisation for low-flow malformations**.** Finally, MRI is essential to define the anatomic extent and involvement of various tissue layers, a distinct advantage over ultrasound [7]. Dynamic CTA—due to its higher temporal resolution—is also a valuable technique for the evaluation of high-flow malformations (Fig. 6; Additional file 2: Video 2). It is important to remember that all vascularised lesions do not necessarily have a vascular origin and that histopathologic analysis remains mandatory in most cases [6, 58–60].

### Vascular malformations

Vascular malformations of the hand are common. They are frequently responsible for a visible mass or an enlarged finger. Low-flow malformations are much more frequent than high-flow malformations [1, 5, 7, 9, 14, 26, 56, 61–64]. They may affect the skin with a colour change (red or blue). The presence of increased warmth, vascular bruit, or thrill suggests a high-flow component.

An AVM is defined by the presence of a nidus. MRA and CTA demonstrate a very rapid lesion enhancement that occurs in the early phases of arterial enhancement, with dilated afferent arteries and early venous drainage through dilated veins. Flow voids are frequently present on conventional MRI sequences (Fig. 7).

Arteriovenous fistula is characterised by a connection between an artery and a vein and a dilation and rapid enhancement of the vein involved in the hyperdynamic circulation (Fig. 6).

Venous and low-flow malformations are uni- or multifocal lesions with variable size that may affect different anatomical structures of different anatomical compartments. These lesions have a micro- or macrocystic pattern and usually a lobulated appearance, appearing hyperintense on fluid sensitive sequences, without flow voids. Phleboliths or thrombi may be present in intralesional venous lakes**.** Afferent arteries are of normal size, and there is no early venous drainage. Contrast enhancement is slow and sometimes very delayed or even absent. On dynamic imaging, the earliest lesion enhancement occurs later than 5 s after arrival of contrast agent in the local arteries [1, 26, 61].

### Vascular tumours

The ISSVA as well as the WHO classification of soft tissue tumours distinguishes between benign vascular tumours, locally aggressive or low metastatic risk intermediate malignant vascular tumours, and malignant vascular tumours [55, 57, 65]. Some of these tumours preferentially affect the hand (Table 5).

**Table 5:** Characteristics of the main vascular and perivascular tumours of the hand according to the International Society for the Study of Vascular Anomalies (ISSVA) classification and the WHO classification of soft tissue tumours

Classification	Tumour	Clinical context	Imaging features	Associated lesions
*Vascular tumour* Benign	Pyogenic granuloma (lobular capillary haemangioma)	Occurs at the site of trauma; occur in up to 5% of pregnancies	Small, exophytic, and hypervascular tumour; increased blood flow	
*Vascular tumour* Benign	Intramuscular haemangioma	Young adults	Lobulated intramuscular hypervascular mass with non-vascular tissue (fat)	
*Vascular tumour* Benign; can evolve to an angiosarcoma	Spindle cell haemangiomas in Maffucci syndrome	Non-hereditary mesodermal dysplasia	Cavernous vascular spaces that sometimes contain phleboliths	Enchondromatosis
*Vascular tumour* Benign	Intravascular papillary endothelial hyperplasia (Masson’s tumour)		Mimics the imaging characteristics of the associated vascular lesions	
*Vascular tumour* Benign	Epithelioid haemangioma	Affects peripheral medium-sized muscular arteries	Hypervascularised tumour developed in the arterial wall	
*Vascular tumour* Borderline	Kaposi's sarcoma	HIV infection	Hypervascularised tumour of the skin	Bone lesions in 85% of the cases
*Vascular tumour* Malignant	Angiosarcoma	Chronic lymphoedema; Maffucci syndrome	Highly aggressive tumour	Chronic lymphoedema; Maffucci syndrome
*Perivascular tumour* Menign (malignant tumours are uncommon)	Glomus tumour	Mostly affects women Painful lesion In the nail bed (up to 90%)	Hypervascularised tumour; less than 1 cm wide; affecting the fingers, usually in the nail bed	
*Perivascular tumour* Benign	Angioleiomyoma	Painful in 50% of the cases	Homogeneous, hypervascularised tumour; less than 3 cm large; in the subcutaneous tissue; may be connected to a vessel	
*Perivascular tumour* Benign	Myopericytoma		Homogeneous and hypervascularised tumour, less than 2 cm large, in the subcutaneous tissue; may be connected to a vessel	

**Fig. 15:**
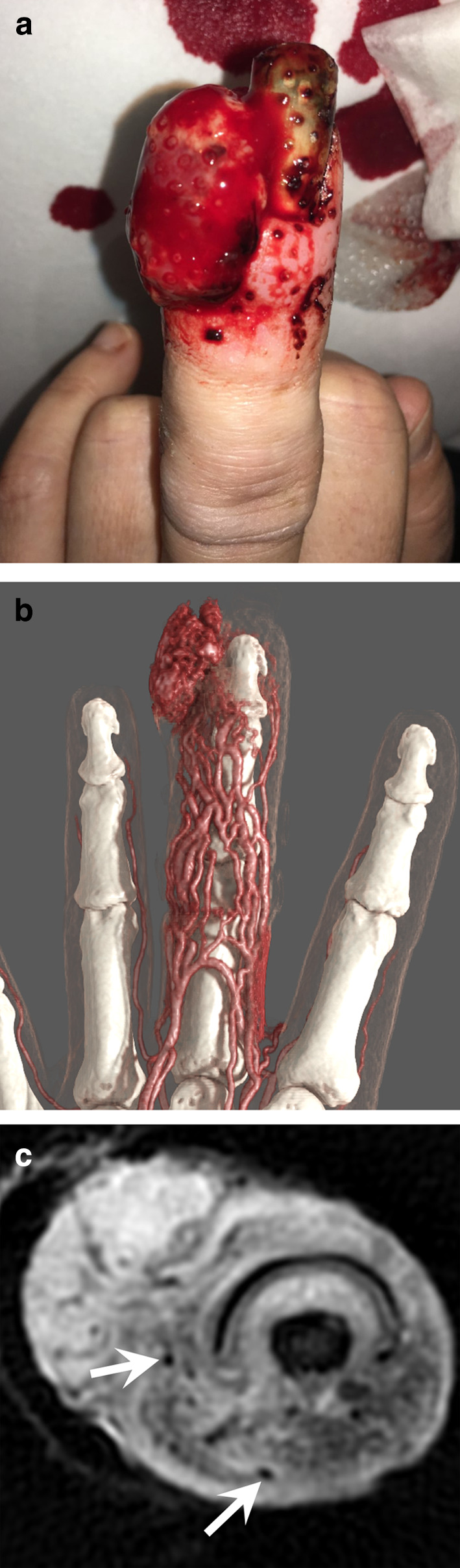
Pyogenic granuloma of the third finger in a 39-year-old woman who recently gave birth. **a** Photograph showing fleshy vascular, haemorrhagic exophytic tumour of the distal phalanx, and an enlargement of the finger. **b** Dynamic CTA showing an increased vascularity of the entire finger associated with a hypervascular exophytic soft tissue tumour. **c** Axial T2-weighted MR image showing an exophytic tumour without clear margins associated with oedema and vessels dilations with flow void (arrows) in the soft tissues

Pyogenic granuloma (or lobular capillary haemangioma) is a common benign tumour, usually diagnosed on the basis of its clinical features, although skin biopsies are required to confirm its nature. Pyogenic granuloma is composed of hyperplastic clusters of capillaries arranged in a lobular architectural pattern. Pyogenic granuloma is a misnomer because the condition is not associated with pyogenic infection and it does not histologically represent a granulomatous inflammation. Therefore, the term ‘lobular capillary haemangioma’ should be preferred. After an initial rapid growth within a few weeks, its size stabilises and rarely exceeds 2 cm. It develops in the superficial dermis—or more rarely in the hypodermis—in the mucosal surfaces, and exceptionally in a vein. The superficial lesions take the appearance of a fleshy vascular, friable, and haemorrhagic nodule. These tumours often occur at the site of even minor trauma, a scratch, or an insect bite. They affect both children and adults. They often accompany pregnancy. The tumour is usually solitary, but disseminated forms exist. Imaging techniques show a small, exophytic, and hypervascular tumour associated with a dramatic increase in the vascular flow of the entire finger (Fig. 15; Additional file 4: Video 4) [66].

**Fig. 16:**
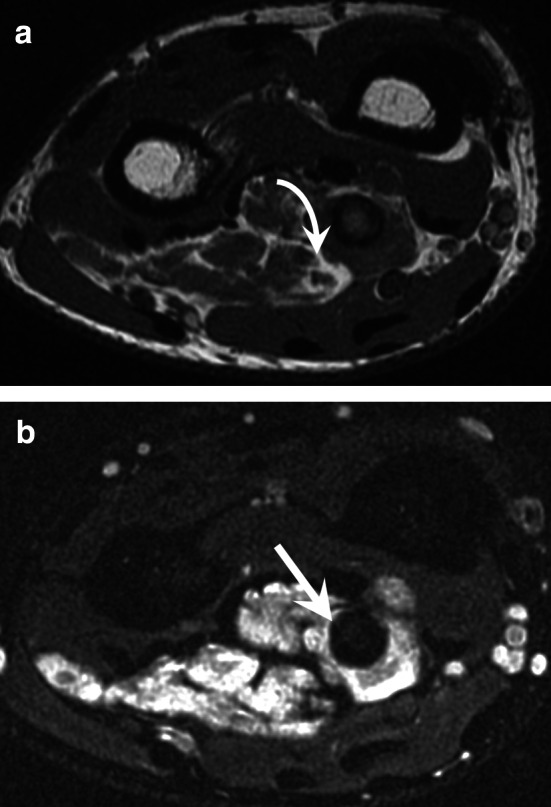
Intramuscular haemangioma in a 41-year-old man. **a, b** Axial T1-weighted (**a**) and T2-weighted (**b**) MR images showing a large mass infiltrating the flexor digitorum profundus muscle, composed of fatty tissue (curved arrow) and vascular lakes containing thrombi and calcifications (straight arrow)

Intramuscular haemangiomas (IMHs) is a rare entity accounting for less than 1% of all haemangiomas. There are still some controversies whether they should be classified as vascular malformations or vascular tumours. They affect adolescents and young adults, appear rapidly, and never regress spontaneously. They are mostly composed of mixed vessel types, including lymphatics, large thick-walled veins, a mixture of cavernous-like vascular spaces and capillaries, or a prominent arteriovenous component [57]. IMH usually appears as a lobulated intramuscular hypervascular mass that also contains mature adipose tissue and sometimes phleboliths, thrombi, or metaplastic ossification (Fig. 16) [67]. These tumours demonstrate an intense enhancement after contrast medium injection, but the vascular flow may be either fast or slow depending upon the vascular composition [67, 68].

**Fig. 17:**
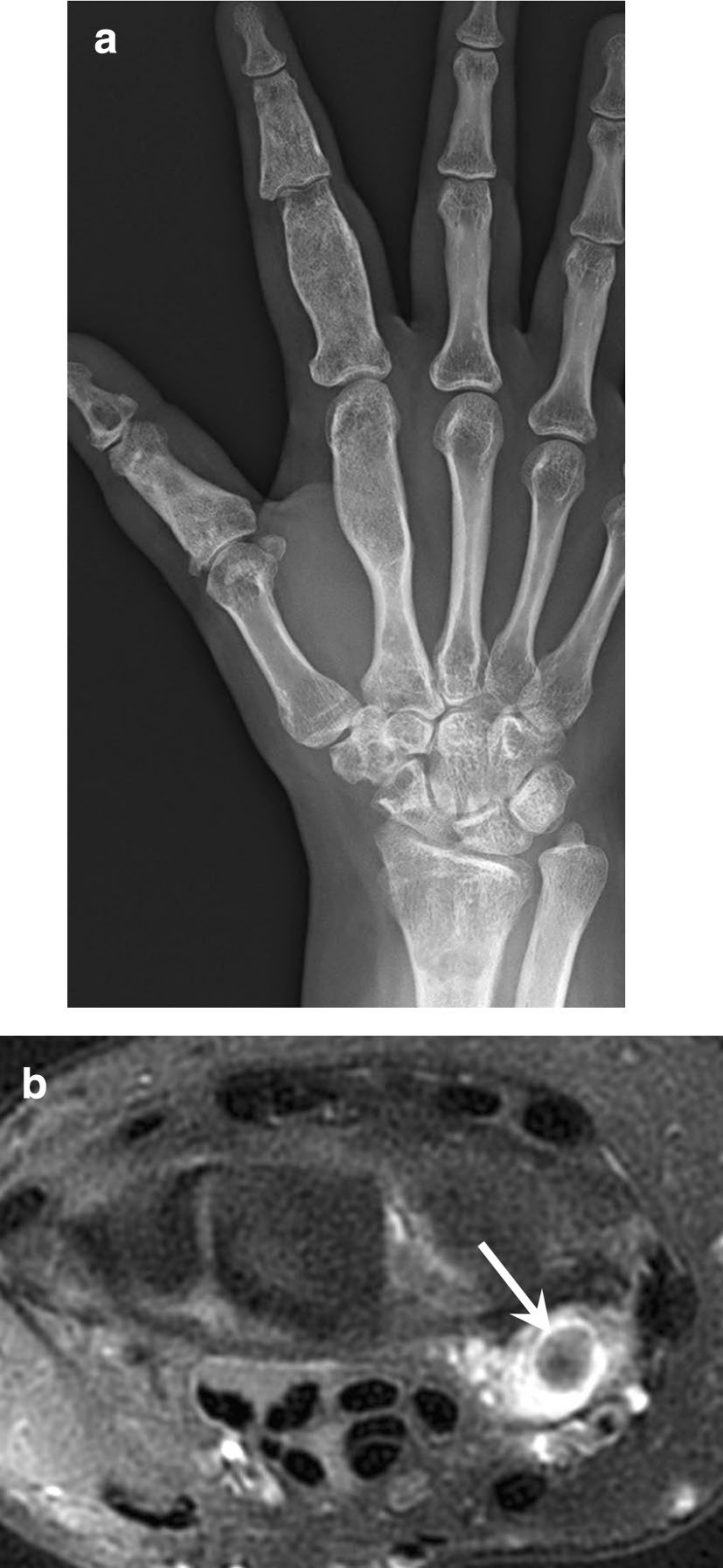
Maffucci syndrome in a 36-year-old woman. (**a**) Standard radiography showing the typical multiple enchondroma of the bones of the hand. (**b**) Axial T2-weighted MR image showing a haemangioma characterised by cavernous vascular spaces containing phleboliths (arrow)

Spindle cell haemangiomas are benign vascular tumours. They present as slowly growing, vascular-appearing, solitary or multiple, dermal or subcutaneous nodules with a predilection for the distal extremities of young adults, especially the hands. Multifocal lesions can be a manifestation of Maffucci syndrome. Maffucci syndrome is a rare, non-hereditary mesodermal dysplasia caused by somatic mutations of the *IDH1* or *IDH2* gene; it is characterised by enchondromatosis associated with spindle cell haemangiomas. The disease begins in childhood with the asynchronous appearance of enchondromas that predominate in the hands and long bones. Haemangiomas appear as small (< 2 cm) lesions that consist of cavernous vascular spaces that sometimes contain phleboliths (Fig. 17). These vascular tumours can evolve into an angiosarcoma in 3%–5% of cases [69].

**Fig. 18:**
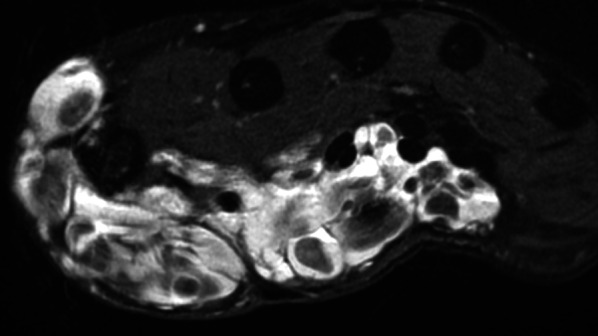
Secondary intravascular papillary endothelial hyperplasia in a 45-year-old woman taking the aspect of a low-flow vascular malformation affecting the thenar eminence. Axial T2-weighted MR image showing a polylobular mass with multiple cavernous lakes containing phleboliths

Intravascular papillary endothelial hyperplasia (IPEH), also called Masson’s tumour, is a benign, reactive, vascular proliferation of endothelial cells that occurs within the vascular lumen associated with an organised thrombus, which may be best described as ‘an exuberant form of organising thrombus’ [70–72]. IPEH constitutes about 2–4% of the benign and malignant vascular tumours of the skin and subcutaneous tissues. IPEH can arise from normal blood vessels (primary IPEH) or in vascular malformations (secondary IPEH). The precise pathogenesis of the disease is still unclear, although it seems to be the response to blood vessel injury or thrombosis. There is no age predilection. MRI of primary IPEH shows lesions usually developed in the digits and in a superficial location, with a high peripheral T2 signal and a variable central T2 signal, a peripheral enhancement (89%), and an associated dominant vessel (73%). [65]. Secondary IPEH mimics the imaging characteristics of the associated vascular lesions (Fig. 18). It is important to correlate imaging findings with histology. Although histological evaluation is the main method for diagnosis, IPEH can histologically simulate angiosarcoma, a malignant tumour with a distinct appearance on MRI [70].

**Fig. 19:**
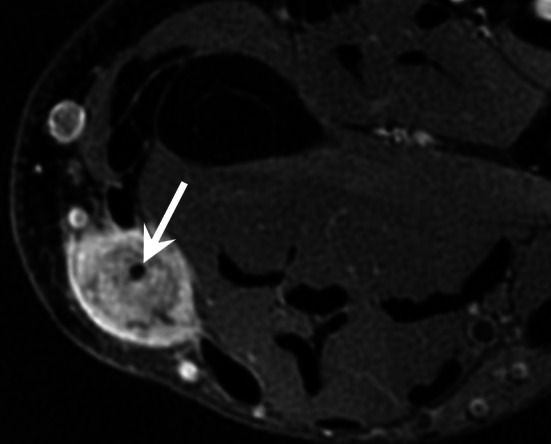
Epithelioid haemangioma of the RA. Axial T2-weighted MR image showing a hypervascular tumour arising from the arterial wall of the RA associated with narrowing of the vessel lumen (arrow)

Epithelioid haemangioma (also called angiolymphoid hyperplasia with eosinophilia, inflammatory angiomatous nodule, or atypical pyogenic granuloma) is a benign vascular tumour reported at diverse anatomic locations including the skin and arteries. These tumours mostly affect men and show a predilection for the extremities. They may also arise within the walls of peripheral medium-sized muscular arteries, such as the UA or RA. MRI usually shows a tumour with focal intramural growth of the affected vessel (Fig. 19). It is noteworthy that these tumours may also mimic a focal arterial aneurysmal dilatation, a malignant vascular neoplasm, or a classic epithelioid sarcoma. Classic (or distal) epithelioid sarcoma is a malignant mesenchymal neoplasm that affects adolescents and young adults. Classic epithelioid sarcoma commonly occurs in the distal upper extremity and affects mainly volar surfaces of the hand, and CT or MRI demonstrates a multinodular mass that extends along vessels, nerves, and fascial planes [57].

**Fig. 20:**
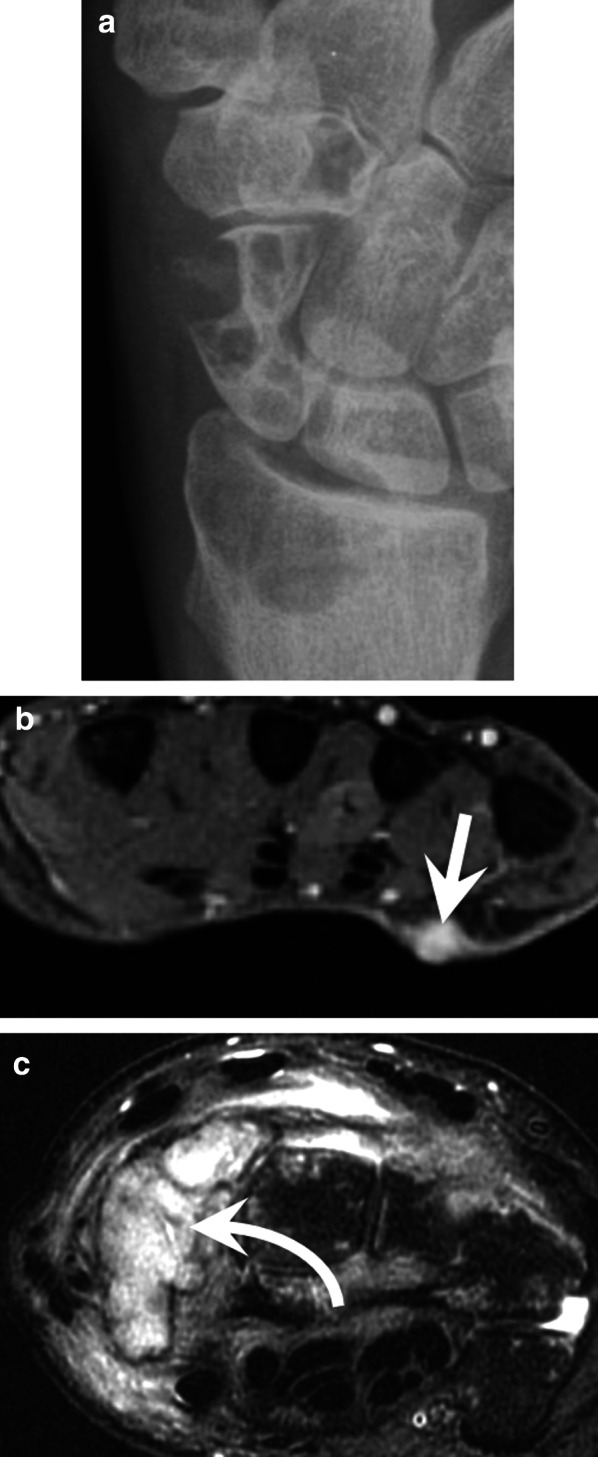
Epidemic Kaposi’s sarcoma in a 40-year-old man. (**a**) Standard radiography showing bone lytic tumours of the radius, scaphoid, and trapezoid bones. (**b**,** c**) Axial T2-weighted MR images showing a small cutaneous tumour of the palm (straight arrow), a tumour invading the scaphoid bone (curved arrow) and soft tissue infiltration

Kaposi’s sarcoma frequently—but not exclusively—affects the skin, where it is the most common sarcoma. It mainly affects adult men. There are four clinical–epidemiological forms: classic Mediterranean, endemic or African, iatrogenic (frequently in transplanted patients), and epidemic (associated with HIV infection). The causative agent is human herpes virus-8 (HHV-8), which has a particular tropism for endothelial cells and is found in all cases. The appearance of skin lesions varies from a simple purplish plaque (sometimes resembling a haematoma) to an ulcerated tumour. Lymphoedema may be present. Aggressive forms are associated with invasion and destruction of the underlying soft tissue and skeleton. CTA and MRA demonstrate hypervascular round or oval nodules with skin thickening. Lytic bone lesions are present in 85% of cases (Fig. 20) [73, 74].

**Fig. 21:**
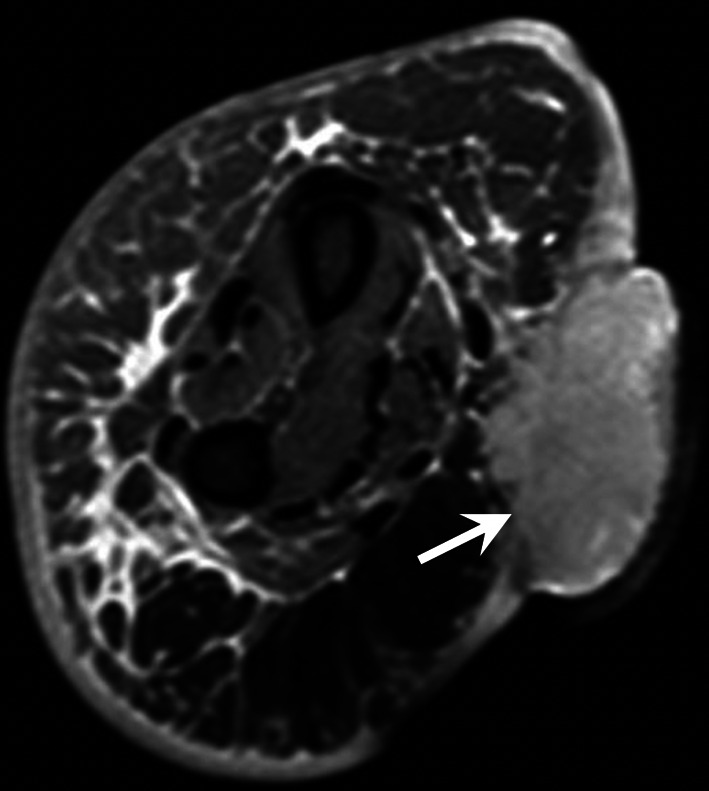
Stewart–Treves syndrome in a 78-year-old woman with a history of mastectomy with axillary lymph node dissection for breast cancer and chronic lymphoedema. Axial T2-weighted MR image showing a skin exophytic tumour developing at the palm of the wrist corresponding to an angiosarcoma (arrow). Note also the subcutaneous infiltration of soft tissue with a honeycomb pattern and the thickening of dermis indicating a chronic lymphoedema

Angiosarcoma is an aggressive, malignant endothelial cell tumour of lymphatic or vascular origin. Angiosarcoma of the hand can be divided into primary cutaneous angiosarcoma and secondary angiosarcoma, associated with chronic lymphoedema (Stewart–Treves syndrome) or radiation therapy (Fig. 21). They may also originate from Maffucci syndrome or haemangioma. MRI shows an irregular, highly vascularised mass with foci of haemorrhage, vessels in the tumour, and invasion of the surrounding tissues. It may also appear as small cutaneous or subcutaneous nodules or take the appearance of a pseudoaneurysm.

### Perivascular tumours

Glomus tumours are rare benign tumours (1%–5% of hand tumours) that arise from the glomus bodies. A glomus tumour is a mesenchymal neoplasm composed of cells resembling the perivascular modified smooth muscle cells of the normal glomus body [57]. The proportion of glomus cells, vascular structures, and smooth muscle tissue makes it possible to define three types: solid glomus tumour (75% of cases), glomangioma, and glomangiomyoma. Glomus tumours also have a nerve component. Malignant glomus tumours are very uncommon [57]. Glomus tumours particularly affect women [75, 76].

Glomus tumours are mainly located in the nail bed (75%–90% of cases), but they can also develop in its matrix, on the side of the fingers, or in the palms of the hands. The average size is 5–6 mm and rarely exceeds 1 cm. Most of them are single, but multiple forms exist (2% of cases).

**Fig. 22:**
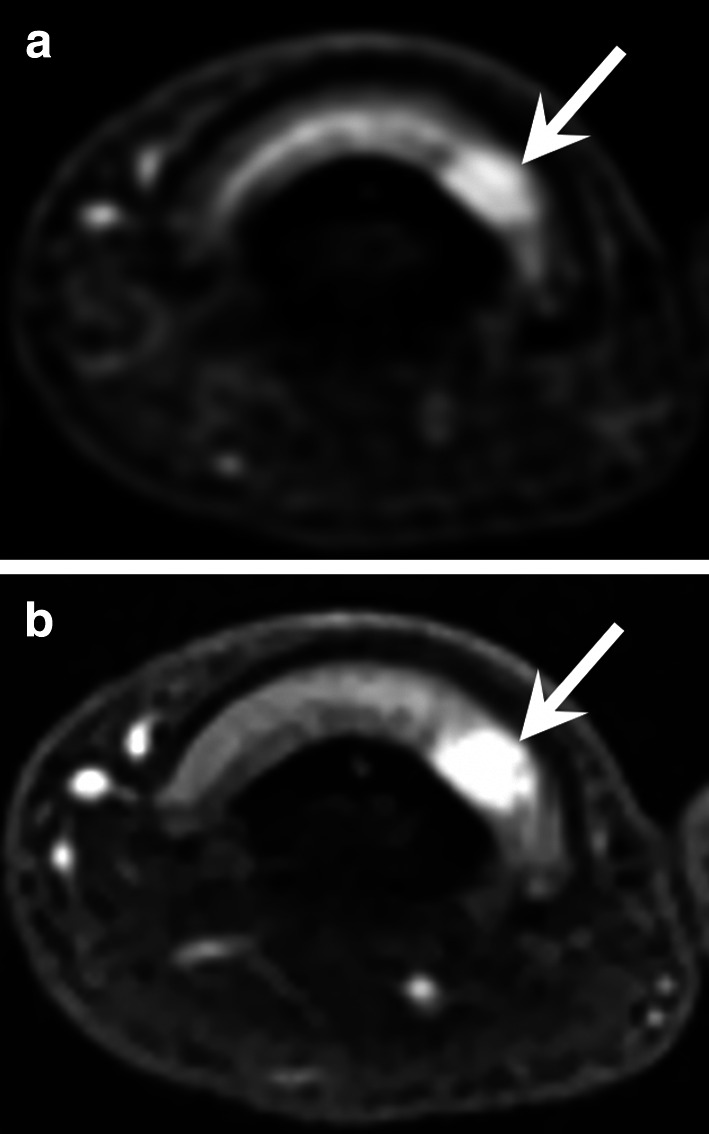
Glomus tumour of the third finger in a 32-year-old woman. **a**,** b** Axial T2-weighted MR images and contrast-enhanced T1-weighted fat-suppressed sequence showing a small hypervascularised nodule (arrow) of the ulnar side of the nail matrix

**Fig. 23:**
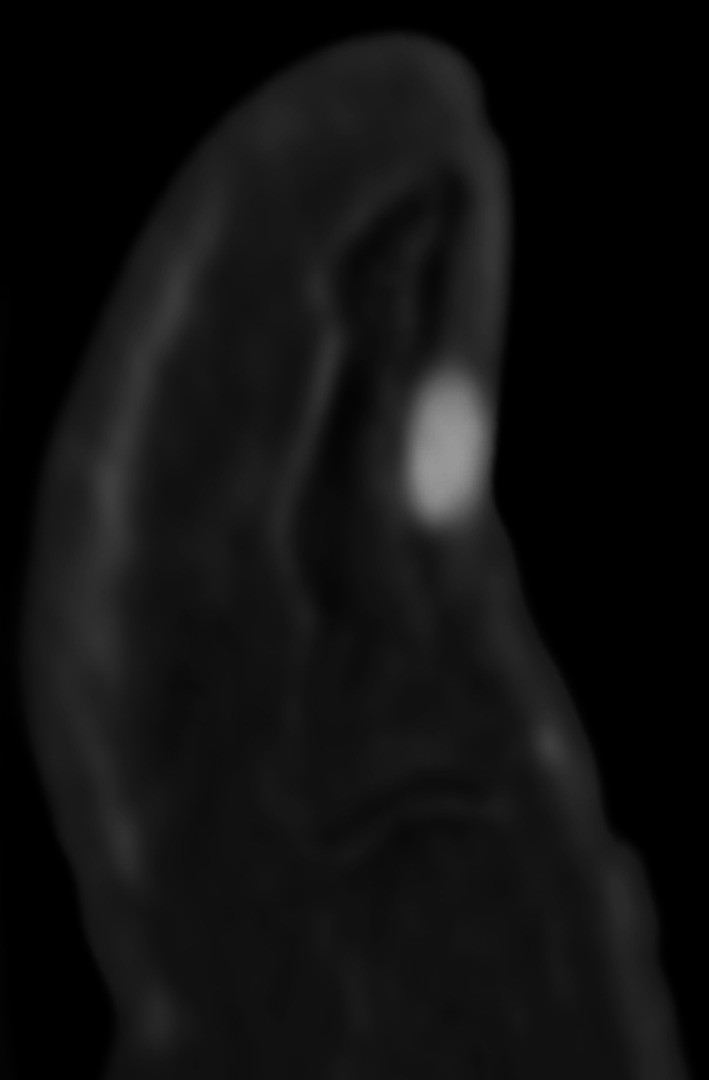
Glomus tumour of the thumb in a 68-year-old man. Dynamic CTA with bone subtraction showing a small hypervascularised tumour (arrow) of the nail bed

Distal lesions of the nail bed are visible as a reddish-blue spot if the lesion is large enough. The typical clinical triad of localised tenderness, severe pain, and cold sensitivity is highly suggestive of a glomus tumour. More proximal sub-matrix lesions are hidden under the posterior skin fold but may result in longitudinal cracking of the nail bed if there is matrix compression [77]. MRI is indicated either as a first-line imaging technique, in particular in the case of painful postoperative recurrence, or after a non-contributory ultrasound examination [6, 63]. MRI can detect lesions as small as 1.5 mm. This examination usually shows a limited nodule, generally homogeneous, hypointense on T1-weighted images and hyperintense on T2-weighted images, hypervascular with a very intense enhancement on MRA, or contrast-enhanced T1-weighted fat-suppressed sequence (Fig. 22) [78, 79]. An erosion of the dorsal surface of the phalanx may be present with larger tumours. Dynamic CTA with bone subtraction also seems to be an efficient tool for the diagnosis of glomus tumours (Fig. 23; Additional file 5: Video 5) [10, 11]. Complete surgical excision is required to ensure the patient achieves complete relief from the symptoms and to avoid recurrence (4%–15% of cases).

Angioleiomyoma is a rare benign vascular smooth muscle tumour arising from the tunica media of vessel walls. Angioleiomyoma affects a wide age range, peaking in the fourth through sixth decades of life. Approximately half of angioleiomyomas are painful. Imaging techniques show a small (less than 3 cm long), well-circumscribed oblong tumour in the subcutaneous fat layer and dermis. The MR pattern is nonspecific with variable enhancement on contrast-enhanced T1-weighted fat-suppressed sequence. However, enhancing vascular structures observed in at least 50% of the cases, on one or both ends of the mass, are suggestive of the diagnosis [80, 81].

Myopericytoma is a rare and distinctive perivascular tumour generally arising in the dermis and subcutis in the distal extremities. Myopericytomas are mostly seen in adults. These tumours are painless. MRI shows a small (less than 2 cm), nodular, well-circumscribed subcutaneous tumour with a strong enhancement on contrast-enhanced T1-weighted fat-suppressed sequence. Myopericytomas are usually connected to a vessel and may develop within the lumen of a vessel.

## Conclusion

Vascular lesions of the hand have various origins. US may be the initial imaging modality, but CTA or MRA is also required, providing detailed vascular mapping, functional information, and precise determination of the extent of the lesions. This information is paramount to guide treatment.

## Supplementary information

**Additional file 1: Video 1**. SHR-CTA of the hand (with GI) in a 69-year-old woman that shows the complex arterial network of the hand with a Type A incomplete superficial palmar arch.

**Additional file 2: Video 2**. Dynamic CTA of an arteriovenous fistula fed by the ulnopalmar digital artery of the fourth finger in a 32-year-old patient.

**Additional file 3: Video 3**. Dynamic CT showing a HHS in a 55-year-old manual worker exposed to different causative factors, including vibrating tools. Note that the delay between each phase is 5 seconds. It takes 35 seconds between the opacification of the RA and the common digital arteries at the level of the metacarpophalangeal joints.

**Additional file 4: Video 4**. Dynamic CTA showing a pyogenic granuloma of the third finger in a 39-year-old woman characterised by increased vascularity of all the finger associated with a hypervascular exophytic soft tissue tumour

**Additional file 5: Video 5**. Dynamic CTA with bone subtraction showing a glomus tumour of the nail bed of the thumb in a 68-year-old man

## Data Availability

Materials described in the manuscript, including all relevant raw data, will be freely available to any scientist wishing to use them for non-commercial purposes, without breaching participant confidentiality.

## Abbreviations

AVM: Arteriovenous malformation
CTA: Computed tomography angiography
DPA: Deep palmar arch
FPMA: First palmar metacarpal artery
GI: Global illumination rendering
HAVS: Hand-arm vibration syndrome
HHS: Hypothenar hammer syndrome
ICM: Iodinated contrast medium
IPEH: Intravascular papillary endothelial hyperplasia
ISSVA: International Society for the Study of Vascular Anomalies
MIP: Maximum intensity projection
MPR: Multiplanar reconstruction
MRA: Magnetic resonance angiography
PAN: Polyarteritis nodosa
RA: Radial artery
SHR: Super-high-resolution
SPA: Superficial palmar arch
SSc: Systemic sclerosis
UA: Ulnar artery
US: Doppler ultrasonography
VRT: Volume rendering technique
